# Common genetic variant rs3802842 in 11q23 contributes to colorectal cancer risk in Chinese population

**DOI:** 10.18632/oncotarget.19702

**Published:** 2017-07-31

**Authors:** Chunze Zhang, Xichuan Li, Weihua Zhang, Yijia Wang, Guanwei Fan, Wenhong Wang, Shuo Chen, Hai Qin, Xipeng Zhang

**Affiliations:** ^1^ Department of Colorectal Surgery, Tianjin Union Medical Center, Tianjin 300121, China; ^2^ Department of Immunology, Biochemistry and Molecular Biology, 2011 Collaborative Innovation Center of Tianjin for Medical Epigenetics, Tianjin Key Laboratory of Medical Epigenetics, Tianjin Medical University, Tianjin 300070, China; ^3^ Department of Pathology, Tianjin Union Medical Center, Tianjin 300121, China; ^4^ First Teaching Hospital of Tianjin University of Traditional Chinese Medicine, Tianjin 300193, China; ^5^ State Key Laboratory of Modern Chinese Medicine, Tianjin University of Traditional Chinese Medicine, Tianjin 300193, China; ^6^ Department of Imaging, Tianjin Union Medical Center, Tianjin 300121, China

**Keywords:** colorectal cancer, rs3802842, meta-analysis, Chinese population

## Abstract

A genome-wide association study identified a common genetic variant rs3802842 at 11q23 to be associated with CRC risk with OR=1.1 and *P* = 5.80E-10 in European population. In Chinese population, several genetic association studies have investigated the association between rs3802842 variant and CRC risk. However these studies reported both positive and negative association results. It is still necessary to evaluate a specific variant in a specific population, which would be informative to reveal the disease mechanism. Until recently, there is no a systemic study to evaluate the potential association between rs3802842 and CRC risk in Chinese population by a meta-analysis method. Here, we aim to evaluate this association in Chinese population by a meta-analysis method using 12077 samples including 5816 CRC cases and 6261 controls. We identified the T allele of rs3802842 to be significantly related with an increase CRC risk (*P*=2.22E-05, OR=1.14, 95% CI 1.07-1.21) in Chinese population.

## INTRODUCTION

Colorectal cancer (CRC) is the third most common type of cancer in the world [1, 2]. CRC is considered to be caused by the interactions between genetic variants and environmental factors [3–6]. In recent years, large-scale genome-wide association studies (GWAS) have identified some novel common CRC genetic variants [7–13]. Tenesa et al. identified a previously unreported common genetic variant rs3802842 on 11q23 to be associated with CRC risk with OR=1.1 and *P* = 5.80E-10 in European population [8].

Evidence shows that allele frequencies, specific linkage disequilibrium structure, and special genetic and environmental backgrounds may cause the risk alleles variation to CRC risk in different populations [14]. Meanwhile, the incidence of CRC is different in populations [15–17]. In Chinese population, several genetic association studies have investigated the association between rs3802842 variant and CRC risk. However these studies reported both positive [18–20] and negative [21–23] association results. It is still necessary to evaluate a specific variant in a specific population, which would be informative to reveal the disease mechanism [14]. Until recently, there is no a systemic study to evaluate the potential association between rs3802842 and CRC risk in Chinese population by a meta-analysis method. Here, we aim to evaluate this association in Chinese population by a meta-analysis method.

## RESULTS

### Study characteristics

In the PubMed database, we got 36 potential studies using the key words ‘rs3802842’ + ‘colorectal cancer’ (up to June 26, 2017). We screened the 36 potential article abstracts, and excluded 20 articles. We further screened the remaining 16 potential full articles, and excluded 11 articles. Meanwhile, we got another one article using Google Scholar database. In the end, we selected six independent case-control association studies in Chinese population [18–23]. All these six studies evaluated the potential association between rs3802842 and CRC risk in Chinese population with a total of 11210 samples including 4794 CRC cases and 6416 controls. All these studies did not depart from Hardy-Weinberg equilibrium. The main characteristics of these six studies are described in Table 1.

**Table 1:** Main characteristics of 6 selected studies in Chinese population

Study	Year	Population	Case #	Control#	*P* value	Case genotype			Control genotype			HWE
						AA	AC	CC	AA	AC	CC	
Xiong [18]	2010	Han Chinese	2124	2124	1.33E-08	640	1052	432	809	963	341	Yes
Ho [21]	2011	Hong Kong Chinese	892	890	0.225	NA	NA	NA	NA	NA	NA	Yes
Li [22]	2012	Han Chinese	229	267	0.771	68	94	45	62	112	33	Yes
Zou [19]	2012	Han Chinese	641	1037	0.000	163	345	133	397	477	163	Yes
Yang [23]	2014	Taiwan Chinese	705	1802	0.133	NA	NA	NA	NA	NA	NA	Yes
Duan [20]	2014	Han Chinese	203	296	0.023	56	94	52	96	153	47	Yes
			4794	6416								

HWE, Hardy Weinberg equilibrium in controls; NA, not available.

### Heterogeneity test

**Figure 1:**
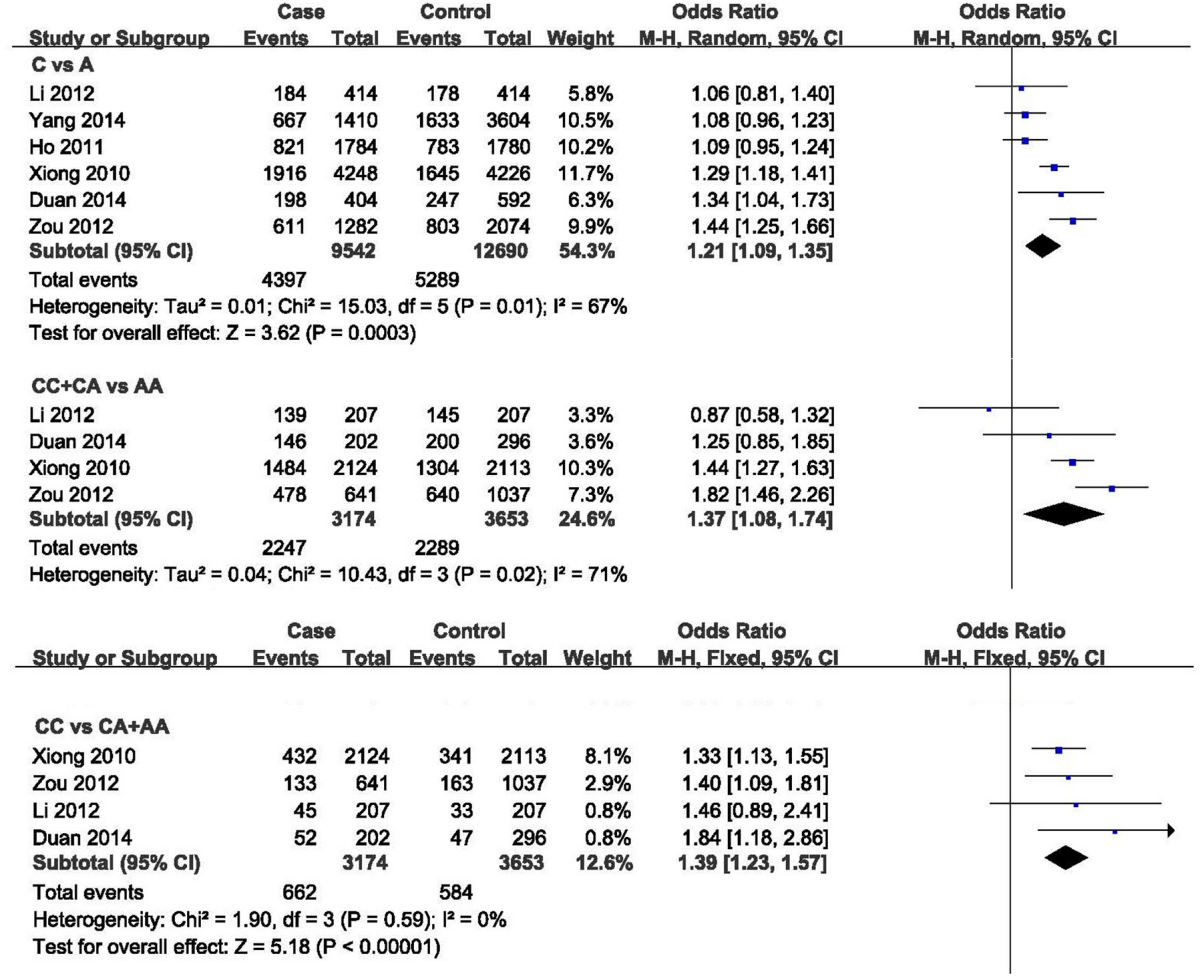
Forest plot for meta-analysis of rs3802842 variant in Chinese population

Using C vs. A model, we identified significant heterogeneity in all the selected six studies with Chi^2^ = 15.03, df = 5 (P = 0.01); I^2^ = 67%. Using CC vs. CA+AA model, we did not identified significant heterogeneity in four of these six studies with Chi^2^ = 1.90, df = 3 (P = 0.59); I^2^ = 0%. Using CC+CA vs. AA model, we identified significant heterogeneity in four of these six studies with Chi^2^ = 10.43, df = 3 (P = 0.02); I^2^ = 71%. The detailed information is described in Figure 1.

### Meta-analysis

In C vs. A model, we applied the random-effect model to perform the meta-analysis, which indicated significant association between rs3802842 C allele and CRC risk with *P*=3.00E-04, OR (odds ratio) =1.21, and 95% CI (confidence interval) [1.09, 1.35]. In CC vs. CA+AA model, we applied the fixed-effect model to perform the meta-analysis, which indicated significant association between rs3802842 CC genotype and CRC risk with *P*=2.22E-07, OR=1.39, and 95% CI [1.23, 1.57]. In CC+CA vs. AA model, we applied the random-effect model to perform the meta-analysis, which indicated significant association between rs3802842 CC+CA genotype and CRC risk with *P*=9.00E-03, OR=1.37, and 95% CI [1.08, 1.74]. The detailed information is described in Figure 1.

### Publication bias analysis

**Figure 2:**
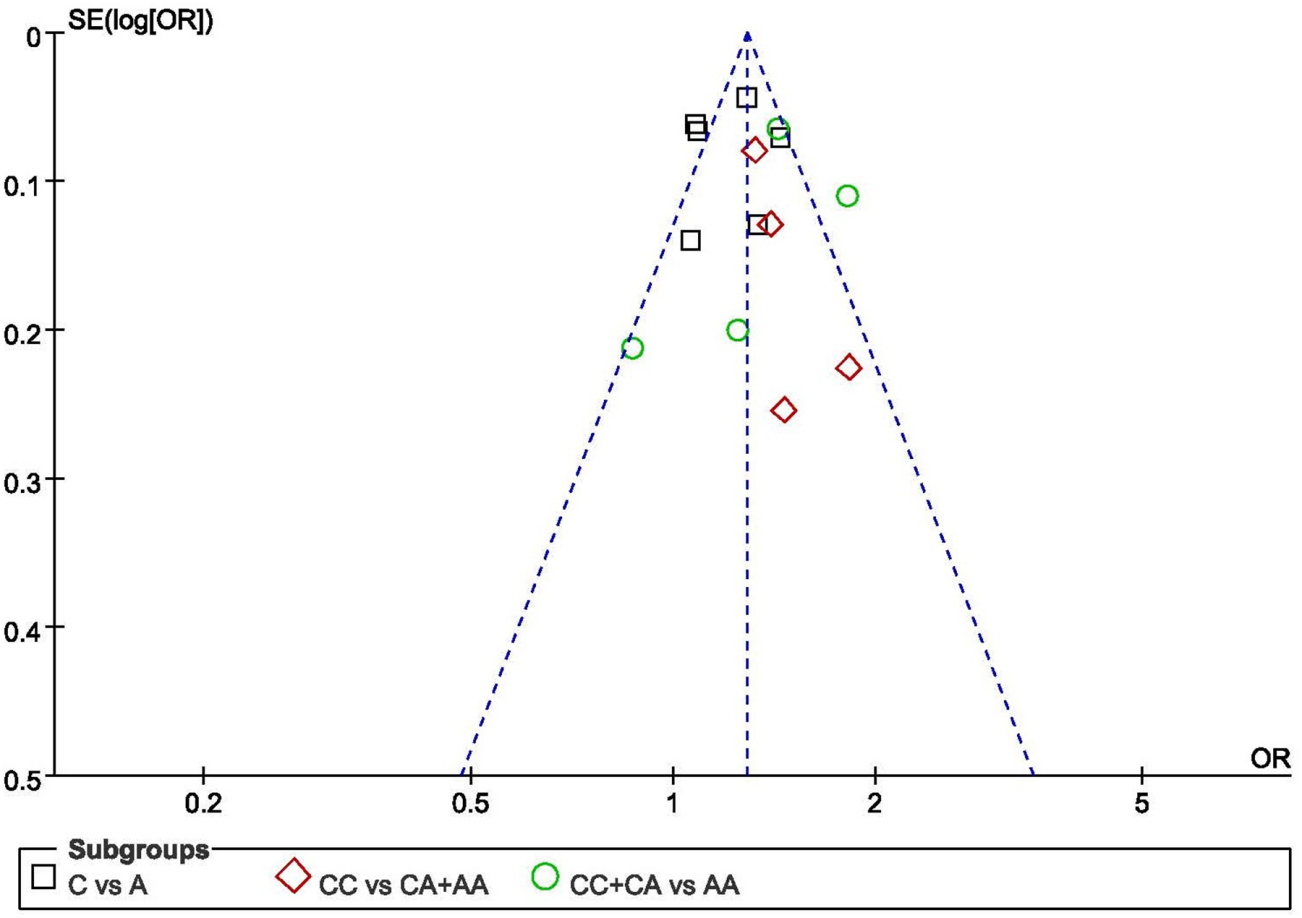
Funnel plot for the recessive model to analyze publication bias of the association of the rs3802842 polymorphism with CRC risk in Chinese population

The possible publication bias of meta-analysis is evaluated by both funnel plot and a regression based statistical approach. Based on the shapes of funnel plots, we did not observe any asymmetric signal in all these three models as described in Figure 2 (Figure 2 illustrates no publication bias for the association of the rs3802842 with CRC risk.). The regression method also did not display any evidence of obvious publication bias with *P*=0.81 for C vs. A model.

### Sensitivity analysis

A leave-one-out sensitivity analysis showed that the pooled ORs were not significantly changed when all these studies were excluded one by one, which indicated that the meta-analysis results were robust and reliable (data not shown).

### Subgroup analysis

In Han Chinese subgroup, we did not identified significant heterogeneity in these four studies with Chi^2^ = 4.21, df = 3 (P = 0.24); I^2^ = 29%. We applied the fixed-effect model to perform the meta-analysis, which indicated significant association between rs3802842 C allele and CRC risk with *P*=9.19E-15, OR=1.31, and 95% CI [1.22, 1.40]. In the combined Hong Kong Chinese and Taiwan Chinese subgroup, we did not identified significant heterogeneity in these four studies with Heterogeneity: Chi^2^ = 0.00, df = 1 (P = 0.98); I^2^ = 0%. We applied the fixed-effect model to perform the meta-analysis, which indicated no significant association between rs3802842 C allele and CRC risk with *P*=0.08, OR=1.08, and 95% CI [0.99, 1.19].

## DISCUSSION

Tenesa et al. identified rs3802842 to be significantly associated with CRC risk [8]. In 2014, Closa et al. analyzed 144 samples and successfully identified that CRC risk loci identified in large-scale GWAS may regulate the expression of nearby genes, which may be candidate targets for developing new strategies for prevention or therapy [24]. Interestingly, rs3802842 in 11q23.1 could significantly regulate the expression of C11orf53, COLCA1 (C11orf92) and COLCA2 (C11orf93) [24]. In 2014, Peltekova et al. analyzed 1,030 CRC cases and 1,061 controls [25]. They also reported COLCA1 and COLCA2 to be regulated by rs3802842 variant [25]. Using tissue microarray analysis, they further showed that rs3802842 was significantly associated with levels of COLCA1 and COLCA2 in the lamina propria [25]. All these findings indicate that rs3802842 is associated with CRC risk and regulate the expression of COLCA1 and COLCA2 genes, which may be involved in pathogenesis of CRC.

Until recently, six independent case-control association studies have been conducted to investigate the association between rs3802842 and CRC risk in Chinese population. Three studies reported positive association results [18–20], and another three studies reported negative association results [21–23]. In this study, we evaluated this association by a meta-analysis using 11210 samples including 4794 CRC cases and 6416 controls, and identified significant association between rs3802842 and CRC in Chinese population.

In our study, we identified significant heterogeneity in these six genetic association studies. We think this may be caused by the substantial genetic variation in Han Chinese population [26]. Chen et al. analyzed 350,000 genetic variants in over 6000 Han Chinese samples from ten provinces of China [26]. Their results showed a one-dimensional “north-south” population structure and a correlation between geography and the genetic structure of the Han Chinese [26].

Considering the significant heterogeneity, we further performed a subgroup analysis in the Han Chinese subgroup, and the combined Hong Kong and Taiwan Chinese subgroup. The results are consistent with previous findings. The heterogeneity in Han Chinese subgroup (I^2^ = 29%) is higher compared with that in combined Hong Kong and Singapore Chinese subgroup (I^2^ = 0%). Meta-analysis further showed the rs3802842 variant to be significantly associated with CRC risk in Han Chinese subgroup, but not in the combined Hong Kong and Taiwan Chinese subgroup.

In 2012, Zou et al. performed a replication study and meta-analysis [19]. In their study, the only selected 4 independent studies in Asian population including 3 independent studies in Chinese population [19]. Here, we selected 6 independent studies in Chinese population to evaluate the association between rs3802842 variant and CRC risk with lager sample size compared with previous study [19]. Our results are consistent with previous findings that there is obvious between-study heterogeneity [19].

## MATERIALS AND METHODS

### Search strategy

**Figure 3:**
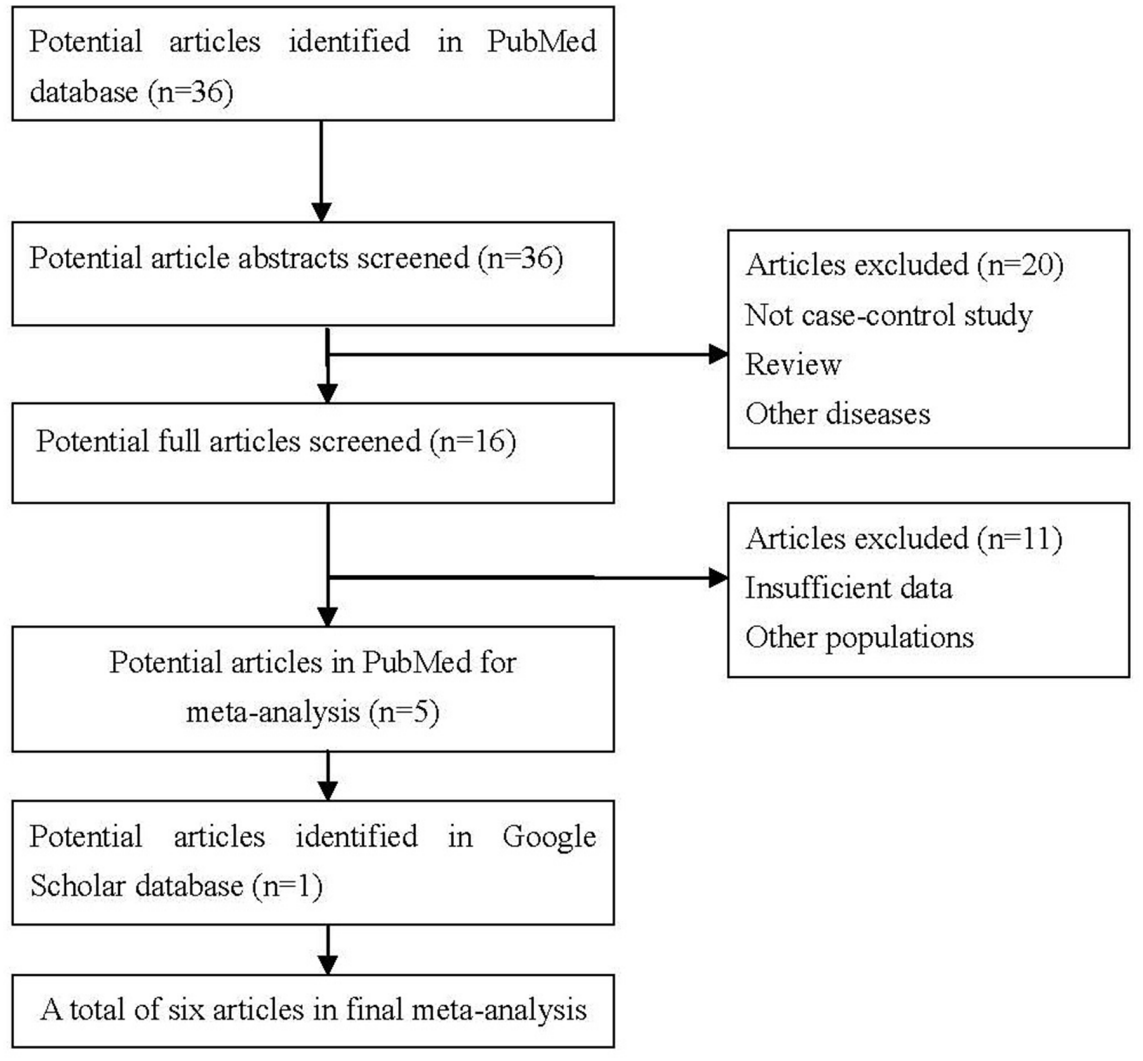
PRISMA flow-diagram showing identification and selection of the pertinent studies for the present meta-analysis

Two reviewers independently selected the potential studies by systematically searching the PubMed database (https://www.ncbi.nlm.nih.gov/pubmed/) using the key words ‘rs3802842’ + ‘colorectal cancer’ (n=36, up to June 26, 2017). We also manually examined additional studies from the references cited in the original literature using Google Scholar database (https://scholar.google.com/), especially all associated publications citing the original CRC GWAS [8]. Here, we limit the following analysis in Chinese population including a native or inhabitant of China or a person of Chinese ancestry. If any two case-control studies overlap with each other, we select the one with the largest sample size in meta-analysis. More detailed information is described in Figure 3, which is a flow diagram of the process used to select eligible studies.

### Study inclusion criteria

The potential genetic association studies should (1) be a case-control design in Chinese population; (2) evaluate the association between rs3802842 and CRC risk; (3) provide the original genotype number, or allele number, or odds ratio (OR) with 95% confidence interval (CI) for one of the three genetic models; or (4) provide sufficient data to calculate the genotype number, or allele number, or OR and 95% CI for one of these three genetic models. We excluded those studies that did not meet the inclusion criteria in following meta-analysis.

### Data extraction

We extracted (1) the name of the first author; (2) the year of publication; (3) the population; (4) the numbers of CRC cases and controls. Two reviewers independently extracted the data carefully. Meanwhile, a third reviewer resolved any disagreement. More detailed information has been widely described in previous studies using the meta-analysis methods [27–44].

### Statistical analysis

In brief, we used Review Manager 5.1 to investigate the potential heterogeneity in all the selected studies by a Cochran's Q test, calculate the pooled OR by a fixed effect model or a random-effect model based on the potential heterogeneity, determine the significance of pooled OR by a Z test.

We calculated the Hardy-Weinberg equilibrium by a chi-square test in R program, if one study provides the control genotype number [45, 46]. If not, we extracted the Hardy-Weinberg equilibrium information from the original studies. Here, three genetic models were selected including C vs. A, CC vs. CA+AA, and CC+CA vs. AA. More detailed information has been widely described in previous studies using meta-analysis methods [27–44, 47, 48].

We investigate potential publication bias by a funnel plot based approach, and a regression based statistical approach proposed by Egger. We performed a sensitivity analysis by a leave-one-out method [49]. We evaluated the influence of each study on pooled OR by omitting each study one at a time [49]. All statistical analyses were performed using Review Manager 5.1 or R, and the significance level is 0.05.

### Subgroup analysis

We performed a subgroup analysis in the Han Chinese subgroup including four studies, and in the combined Hong Kong Chinese and Taiwan Chinese subgroup including two studies using C vs. A model.
